# Progress Made in Non-Metallic-Doped Materials for Electrocatalytic Reduction in Ammonia Production

**DOI:** 10.3390/ma17102419

**Published:** 2024-05-17

**Authors:** Gerald D. S. Quoie Jr, Mingshuo Jiao, Krisztina Lászlód, Ying Wang

**Affiliations:** 1 State Key Laboratory of Pollution Control and Resources Reuse, College of Environmental Science and Engineering, Tongji University, Shanghai 200092, China; gerald4991@outlook.com (G.D.S.Q.J.); 2152659@tongji.edu.cn (M.J.); 2 Shanghai Institute of Pollution Control and Ecological Security, Shanghai 200092, China; 3 Department of Physical Chemistry and Materials Science, Budapest University of Technology and Economics, H-1521 Budapest, Hungary

**Keywords:** electrocatalysis, nitrate reduction, ammonia, non-metallic-doped catalysts, defect engineering, biomass-derived carbon

## Abstract

The electrocatalytic production of ammonia has garnered considerable interest as a potentially sustainable technology for ammonia synthesis. Recently, non-metallic-doped materials have emerged as promising electrochemical catalysts for this purpose. This paper presents a comprehensive review of the latest research on non-metallic-doped materials for electrocatalytic ammonia production. Researchers have engineered a variety of materials, doped with non-metals such as nitrogen (N), boron (B), phosphorus (P), and sulfur (S), into different forms and structures to enhance their electrocatalytic activity and selectivity. A comparison among different non-metallic dopants reveals their distinct effects on the electrocatalytic performance for ammonia production. For instance, N-doping has shown enhanced activity owing to the introduction of nitrogen vacancies (NVs) and improved charge transfer kinetics. B-doping has demonstrated improved selectivity and stability, which is attributed to the formation of active sites and the suppression of competing reactions. P-doping has exhibited increased ammonia generation rates and Faradaic efficiencies, likely due to the modification of the electronic structure and surface properties. S-doping has shown potential for enhancing electrocatalytic performance, although further investigations are needed to elucidate the underlying mechanisms. These comparisons provide valuable insights for researchers to conduct in-depth studies focusing on specific non-metallic dopants, exploring their unique properties, and optimizing their performance for electrocatalytic ammonia production. However, we consider it a priority to provide insight into the recent progress made in non-metal-doped materials and their potential for enabling long-term and efficient electrochemical ammonia production. Additionally, this paper discusses the synthetic procedures used to produce non-metal-doped materials and highlights the advantages and disadvantages of each method. It also provides an in-depth analysis of the electrochemical performance of these materials, including their Faradaic efficiencies, ammonia yield rate, and selectivity. It examines the challenges and prospects of developing non-metallic-doped materials for electrocatalytic ammonia production and suggests future research directions.

## 1. Introduction

Ammonia (NH_3_) is a highly significant chemical compound in contemporary life and is extensively used in agriculture, pharmaceuticals, textiles, and various other industries [1,2,3,4,5]. Due to its high energy density (4.32 kW h kg^−1^), high hydrogen content, and zero carbon emissions, it is an excellent energy carrier conducive to carbon neutrality [6,7]. The Haber–Bosch ammonia synthesis process, developed in 1905, remains the primary method for ammonia production. This process requires harsh conditions, such as reaching temperatures of 400–500 °C and pressures of 150–300 atm [8,9]. It is associated with significant energy consumption, accounting for 1–2% of global energy usage, and contributes to over 1% of the total CO_2_ emission [10,11]. The exploration of non-metallic-doped materials for electrocatalytic reduction in ammonia production has witnessed significant advancements in recent years, reflecting the quest for sustainable and efficient nitrogen fixation processes [12,13,14]. Early studies, such as those by Humphrey Davy [15], Martín et al. [16], Fitchter and Suter [17], Tamelen et al. [18], Ren et al. [19], Tsumeto et al. [20], Rod et al. [21], Kordali et al. [22], Köleli et al. [23], Skulason et al. [24], Azofra et al. [25], Abghoui and Skúlason [26], Zhao et al. [27], and Du et al. [28], laid the groundwork by demonstrating the electrocatalytic capabilities of nitrogen reduction reactions (NRRs) for NH_3_ synthesis.

Therefore, electrochemistry is an effective method for studying redox processes. The electrochemical hydrogenation reduction of N_2_ to synthesize NH_3_ is feasible at room temperature, circumventing the need for hydrogen with high energy density. Furthermore, the electrochemical reduction approach is capable of efficiently treating nitrate-laden wastewater, facilitating an eight-electron transfer to yield NH_3_ [29,30]. Owing to its reduced energy demand and enhanced Faradaic efficiency, this methodology has received increasing scholarly attention [31,32,33]. The bond enthalpy of N=O in nitrate is substantially lower (204 kJ/mol) than that of the N≡N triple bond (941 kJ/mol), indicating that ammonia production via nitrate reduction is more energy-efficient than traditional ammonia synthesis via nitrogen hydrogenation [34,35,36].

Additionally, this technique provides a novel remedial strategy for aquatic nitrate pollution, offering concurrent advantages for environmental conservation and economic progress [37]. Under high overpotential conditions, electrocatalytic reactions are concurrent with a pronounced hydrogen evolution reaction (HER), which affects the Faradaic efficiency (FE) [38]. Moreover, given the diverse oxidation states of nitrogen, the reduction pathway may produce multiple side products, thereby attenuating the yield [39]. Consequently, the utilization of high-performance catalysts to amplify reaction kinetics and selectivity is of paramount importance [40,41]. Non-metallic doping is a viable approach to bolster the activity of catalysts.

Despite significant advancements in the field of electrocatalytic reduction for ammonia production, there is still a need for a comprehensive understanding of how non-metallic-doped materials enhance catalytic efficiency. Although there have been many investigations into the use of non-metallic catalysts, there is a relative lack of exploration of the potential advantages and mechanisms of non-metallic doping. This study gap emphasizes the need for additional investigations to clarify the fundamental principles governing the electrocatalytic activity of non-metallic-doped materials and their influence on the efficiency of NH_3_ production. Therefore, we present a thorough examination of advancements in the utilization of non-metallic-doped materials for electrocatalytic reduction in the production of NH_3_. Through our investigation of this research void, our objective was to provide insight into the fundamental mechanisms, improvements in performance, and potential future development of non-metallic-doped catalysts for the advancement of sustainable NH_3_ synthesis methods.

## 2. Construction Principle of Non-Metallic-Element-Doped Catalyst

**Figure 1:**
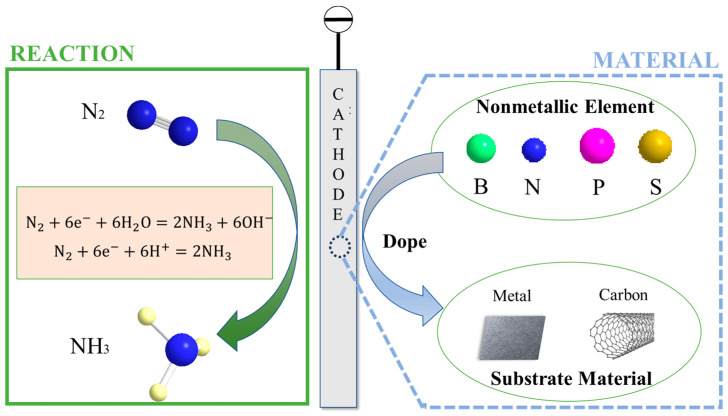
Non-metallic-element-doped material schematic.

Various concepts guide the construction of non-metallic-doped catalysts, with the aim of enhancing the electrocatalytic activity by modifying its structure and characteristics. Figure 1 depicts the doping of non-metallic elements like boron (B), nitrogen (N), phosphorus (P), and sulfur (S) into metal-based or carbon-based electrodes.

**Figure 2:**
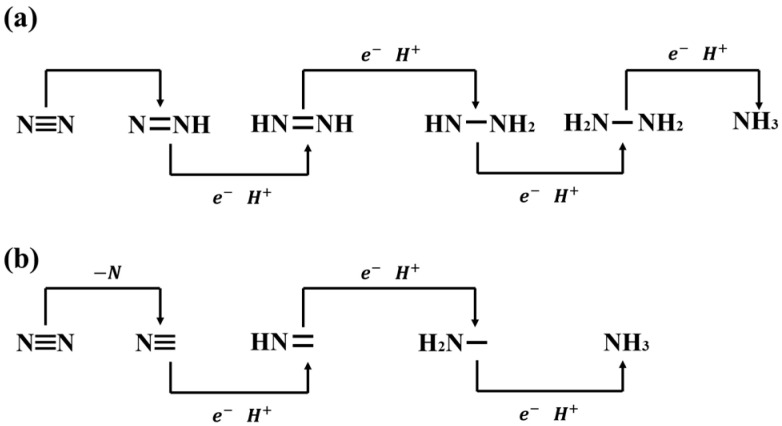
(**a**). The first possible electrocatalytic pathway for nitrogen to produce ammonia. (**b**) The second possible electrocatalytic pathway for nitrogen to produce ammonia.

Multiple proton reduction steps characterize the process of ammonia production through reduction. In the pathway of nitrate reduction, the rate-determining step is the reduction of nitrate to nitrite [42]. In the case of nitrogen reduction, there are two main pathways: one involves breaking the nitrogen-to-nitrogen bond and, in the final step, concurrently producing ammonia as the end product (Figure 2a), while the other consists of breaking the nitrogen-to-nitrogen bond in the first step (Figure 2b) [43].

### 2.1. Defect Engineering

Defect engineering has been widely used to improve electrocatalytic performance [44]. This is fundamental for the development of non-metallic catalysts for NH_3_ synthesis. Non-metallic materials are incorporated to alter the structure and surface characteristics of the catalyst and introduce imperfections [45]. Defect engineering pertains to the deliberate introduction of imperfections or aberrations into the catalyst structure. These flaws may contain edges, voids, or dislocations that can act as catalytic reaction sites [46]. Adding these flaws increases the reactivity and surface area, improving the electrocatalytic effectiveness of reactions such as the reduction of nitrate (NO_3_^−^) to NH_3_. The most commonly employed non-metallic elements for catalyst doping in NH_3_ production include B, N, P, and S [47]. These elements are preferred because of their ability to adjust the catalyst surface characteristics and electronic structure, thereby enhancing the catalytic activity for NH_3_ production. Despite the challenges and limitations related to defect control and stability, defective non-metallic catalysts hold significant promise for sustainable and efficient catalytic applications.

Huang et al. made g^−^C_3_N_4_, a porous polymeric carbon nitride with a controllable number of nitrogen vacancies (NV), by calcining the material in two easy steps. This study found that introducing NV can significantly enhance the electrocatalytic reduction performance of g^−^C_3_N_4_ for nitrate, achieving a nitrate removal rate of 89.1% and high Faraday efficiency while also exhibiting high NH_3_ selectivity [48]. Researchers have constructed reduced graphene oxides via defect engineering, where carbon vacancies facilitate strong binding with N_2_ while suppressing H binding. This approach has achieved high Faradaic efficiencies (up to 22.0%) across a wide pH range [49].

### 2.2. Post-Synthesis Doping Strategies

Post-synthetic doping approaches involve direct interactions between a carbon material that has already been synthesized and sources containing nitrogen, such as urea, NH_3_, dicyandiamide (C_2_H_4_N_4_), dimethylformamide (C_3_H_7_NO), and melamine (C_3_H_6_N_6_) For example, the incorporation of nitrogen into the carbon network can be facilitated by high temperatures, prolonging the reaction periods, adding transition metals, or preparing a carbon surface [50]. Although these methods have some disadvantages, they are widely used because of their simplicity in producing N-doped carbons. The nitrogen content and arrangement were affected by the temperature and time of the process. [51]. While some studies indicate that NH_3_, as a precursor, shows potential at 500–600 °C, other studies propose temperatures exceeding 900 °C for peak nitrogen content with NH_3_ [52].

Post-synthetic doping methods involve a range of techniques, such as microwave-assisted carbonization, which leads to the production of N-doped reduced graphene oxide (N-RGO) containing pyridinic, pyrrolic, and graphitic nitrogen groups [53,54]. Urea is another nitrogen-containing precursor that is known for its low pyrolysis temperature and low cost. For instance, N-doped carbon with 4% nitrogen content was later treated with urea, increasing its nitrogen content to 11.08% and enhancing its adsorption characteristics [55]. Therefore, the modification of carbon compounds by the deposition and subsequent carbonization of N-containing precursors is a noteworthy approach.

Several approaches have demonstrated efficacy. Chen et al. [56] employed hydrazine (N_2_H_4_) and NH_3_ to decrease the size of graphene oxide (GO) at temperatures lower than 200 °C, resulting in the production of nitrogen-doped graphene sheets (NG) with a nitrogen content of 5%. The polymerization and carbonization of aniline produce N-doped carbon fibers [57]. Innovative methods, such as the tour technique employing aryldiazonium salts, enable the tuning of N-functionalities in carbon nanotubes (CNTs) or graphene [58]. Electrochemical functionalization with aminobenzene acids yields N-modified CNTs with various nitrogen and oxygen groups [59].

Nitric acid treatment, either as a pretreatment or for chemical reactions with nitrogen-containing compounds, has been effective in diverse synthesis approaches [60,61]. The utilization of nitric acid and amine functionalization has been demonstrated to be successful in achieving the solubilization of single-walled CNTs. This technique involves the hydrolysis of N-doped carbon at elevated temperatures [62]. Lu et al. [63] demonstrated the adaptability of nitric acid as a pretreatment for various N-doped carbon compounds for the oxygen reduction reaction (ORR). They specifically employed nitric acid and melamine to create N-doped-ordered mesoporous carbon.

Table 1 summarizes several post-synthesis methods, showing the enhanced characteristics of doped materials employing non-metals, such as N, B, carbon (C), and silicon (Si) from various sources across different materials.

Chemical vapor deposition (CVD) has been employed to create co-doped microporous carbons with Y-zeolite infiltration, revealing predominant pyridinic, pyrrolic, and graphitic nitrogen [70]. CVD with C_2_H_4_/NH_3_ combinations also successfully produced nitrogen-doped carbon nanofibers (N-CNFs) with enhanced pyridinic group content. Carbonization uses a variety of nitrogen-containing sources, including conducting polymers, ionic liquids, and biomass, to produce N-doped carbons with various topologies and nitrogen concentrations [71,72].

For instance, Gavrilov et al. [73] produced N-doped carbon materials by carbonizing polyaniline (PANI) at a temperature of 800 °C. This process resulted in a nitrogen content of approximately 9%, mostly comprising pyridinic and graphitic groups. The N-doped carbon compounds obtained from PANI exhibited varied proportions of graphitic and pyridinic groups depending on the experimental conditions. This highlights the impact of factors such as carbonization temperature, monomer type, and polymerization length [74]. Imidazolium and pyridinium ionic liquids have also been used to produce mesoporous N-doped carbon with a nitrogen content exceeding 15% by weight [75]. Moreover, the distinctive characteristics of Metal–Organic Frameworks (MOFs) containing N-organic ligands have prompted investigations into their potential as precursors for N-doped carbon materials [76].

Furthermore, in situ techniques involving biomass, particularly hydrothermal carbonization (HTC), offer an eco-friendly route. Nevertheless, hydrochars typically require supplementary procedures, such as heat treatment or chemical activation, to improve their surface area, which may result in a reduction in the amount of nitrogen present [71]. The traditional decomposition of biomass without chemical activation effectively generates N-doped carbon nanosheets with a high surface area from chitosan [77]. A novel method utilizing KCl/ZnCl_2_ to combine biomass and melamine resulted in the production of mesoporous N-doped carbons with a significant nitrogen concentration of 11.9% [78]. Despite the obstacles in regulating nitrogen speciation and content, the synthesis of N-doped carbon materials by diverse in situ processes has been significant.

### 2.3. In Situ Doping Methods

As seen in Table 2, in the context of in situ doping synthesis, simultaneous carbon material synthesis and nitrogen doping are achieved, with CVD being a widely utilized technique, particularly for graphene, CNTs, CNFs, and microporous carbon production [79]. Several researchers have devised novel techniques for manufacturing co-doped microporous carbons with large surface areas. The process involves impregnating the Y zeolite with 1-ethyl-3-methylimidazolium tetracyanoborate, an ionic liquid containing nitrogen, and chemical vapor deposition (CVD). This method results in the formation of microporous carbon co-doped with nitrogen in the form of pyridinic, pyrrolic, and graphitic nitrogen [70].

A wide range of N-containing sources, including conducting polymers, ionic liquids, and biomass, have been utilized for carbonization to produce nitrogen-doped carbons with different structures and N levels. Lu et al. [82] used electrospinning to create hollow-structured N-doped carbon nanofibers (N-CNFs) with significant graphitic characteristics by combining PAN and ZIF-8. These nanofibers achieved a nitrogen content of 8% after carbonization at 900 °C. The N-containing groups in the final N-CNFs primarily consisted of pyridinic and pyrrolic groups, with the latter changing to a graphitic form at elevated carbonization temperatures [77].

Hydrothermal carbonization (HTC) is an eco-friendly and cost-efficient method for producing N-doped carbon compounds [83]. Although in situ techniques involving biomass, mainly through hydrothermal carbonization (HTC), offer an eco-friendly route, the resulting hydrochars often require additional steps for heat treatment or chemical activation to enhance the surface area and deplete some nitrogen content [71]. Chitosan was successfully converted into N-doped carbon nanosheets with large surface areas using traditional biomass pyrolysis without chemical activation [84].

## 3. Regulation of Nitrogen Reduction by Non-Metallic-Doped Catalysts

The foundational element in crafting catalysts doped with non-metallic materials, such as N-CNTs, for NH_3_ synthesis is the integration of non-metallic elements, such as B and N, to create active sites. For instance, incorporating B-N prevents undesired HER while providing ample active sites on carbon nanosheets. Utilizing non-metallic materials, mainly two-dimensional nano-non-metallic materials with doped heteroatoms, has significantly advanced eco-friendly and sustainable synthesis [85].

### 3.1. Boron-Doped (B-Doped) Catalysts

B-doped catalysts, particularly B-N-doped materials, exhibit notable potential as active sites for the electrocatalytic and photocatalytic reduction of N_2_ to NH_3_ [86,87]. These materials offer sufficient active sites for NH_3_ generation while effectively suppressing competing hydrogen evolution processes, making them advantageous for targeted electrocatalytic nitrogen reduction [88]. However, NH_3_ synthesis using B-doped catalysts involves the electrocatalytic reduction of NO_3_^−^ to NH_3_. The B-doped catalyst facilitates redox reactions, converting nitrate to nitrite by transferring electrons from the catalyst to nitrate [89,90]. The adsorption of nitrite on the catalyst surface leads to the adsorption of N species, which are highly reactive and easily reduced to NH_3_ [91,92].

Further reduction of the adsorbed N species on the B-doped catalyst produced ammonia. This process involves the gain of electrons from nitrogen species, which interact with hydroxyl groups on the catalyst surface to form NH_3_. The essential role of the B-doped catalysts lies in facilitating these redox processes, lowering the energy barrier for nitrogen reduction, and enhancing the overall reaction rate. The synthesis concluded that the generated ammonia was desorbed from the catalyst surface. The unique structure and electrical characteristics of the B-doped catalysts contribute to efficient NH_3_ desorption, yielding significant amounts of NH_3_ [93].

Ongoing research has explored the impact of boron concentration on B-doped catalysts in NH_3_ synthesis, revealing significant effects on electrocatalytic and photocatalytic nitrogen reduction [47]. The boron content of B-doped carbon nanosheets influences the efficiency and selectivity of the electrocatalytic nitrogen reduction to ammonia [94]. In electrocatalytic ammonia generation, graphene with a boron doping concentration of 6.2% demonstrated superior faradaic efficiencies and NH_3_ yields [95].

**Figure 3:**
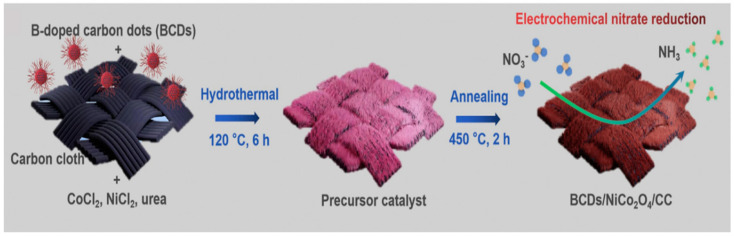
Schematic illustration of BCDs/NiCo_2_O_4_/CC nanowire array preparation. Adapted with permission from Lu et al. [89]. Copyright 2022, ScienceDirect.

The dispersion of distinct boron structures in boron-doped graphene impacts electrocatalytic nitrogen reduction, with a higher ratio indicating an increased catalytic activity. The type of catalyst material and the boron concentration collectively influence the specific catalytic activity [96]. The lower boron concentration in the B-doped Ni/SBA-15 catalysts reduces their catalytic activity [97]. Boron clusters (BC_3_) in B-doped graphene serve as significant electrocatalysts for the nitrogen reduction reaction (NRR) sites, enhancing graphene reactivity and affecting surface oxidation and catalytic activity [98]. A typical synthesis method for B-doped materials is shown in Figure 3.

### 3.2. Nitrogen-Doped Catalysts

N-doped catalysts, including B-doped variants, are now increasingly employed in the electrocatalytic reduction of NO_3_^−^ to NH_3_ because most undoped electrocatalysts show low efficiencies and NH_3_ yields, primarily because of the non-polar nature and high binding energy (940.95 kJ/mol) of the triple bond of N molecules, making it difficult to break and slow down the NRR [89]. These doped catalysts generate active sites that cleave the N-N bond, enabling N_2_ adsorption and the subsequent activation of nitrogen atoms for reduction. The resulting adsorbed nitrogen species are highly reactive and readily reduced to ammonia [99]. N-doped catalysts facilitate electron gain from N species and interactions with hydroxyl groups, thereby accelerating the reduction process. The synthesis ended with NH_3_ desorption from the catalyst surface, yielding a high output. N-doped phosphorene has shown exceptional performance in this process [100].

Table 3 provides a concise summary of the different synthetic techniques used to prepare the metal catalysts supported on N-doped carbon materials. These methods include the use of capping agents to regulate the formation of nanoparticles, nitrogen-doped carbon to ensure a uniform dispersion of nanoparticles, and MOFs as sacrificial templates for creating metal/N-carbon compositions with distinct properties. For example, N-doped carbon has emerged as a compelling alternative for supporting nanoparticles (NPs) and promoting small and well-dispersed NPs, thereby enhancing catalytic performance [101]. Although capping agents have historically regulated NP growth, their impact on catalytic activity is debatable. N-doped carbon supports are promising alternatives for overcoming the limitations associated with capping agents [102]. Nitrogen functionalities in N-doped carbon act as “tethers”, aiding NP dispersion and conferring resistance to agglomeration and coarsening, thus enhancing catalytic performance [103].

Existing research demonstrates that nitrogen functional groups on carbon supports have a significant impact on the deposition of NPs, the interaction between the metal and support, the electrical properties of the NPs, and the acid–base properties of the resulting materials. Several synthetic methods have been investigated to control the nucleation, growth, and stabilization of N-containing carbons in nanoparticles [104]. The synthesis of NPs supported on N-doped carbon materials can be divided into three main approaches: (i) deposition of NPs onto pre-existing N-doped carbon materials, (ii) simultaneous integration of nitrogen and metal phases onto pre-existing carbon materials, and (iii) on-site formation of NPs on N-doped carbon supports [106].

Typically, methods such as impregnation, deposition–precipitation, and sol immobilization are used to load nanoparticles onto pre-synthesized N-doped carbon materials. These methods control the properties of the support material; however, they require numerous processes [105]. The process of synthesizing NPs on carbon supports doped with nitrogen, undertaken through a pyrolysis stage, enables the simultaneous integration of metal and nitrogen functionalizations. This incorporation occurs under certain conditions and affects the features of the NPs [107]. One notable method involves the use of MOFs as sacrificial templates, in which metal joints and organic linkers undergo precision pyrolysis to transform them into carbon metal–carbon composites with nitrogen. This approach produces catalysts with fascinating compositions and structures, such as gold nanoparticles embedded in N-doped nanoporous carbon, cobalt nanoparticles in N-doped porous carbon, and various other configurations [110].

N-phosphorene was produced by combining N-methyl-2-pyrrolidone (NMP) solvent with ball milling and microwave-assisted exfoliation. Bulk BP crystals were initially ground to increase the surface reactivity [111]. Subsequently, the materials were ball-milled in NMP with or without NH_4_OH, resulting in the production of N-doped or O-phosphorene. Ball milling weakens the van der Waals contacts and produces reactive species at nanosheet boundaries [108]. Ultrathin phosphorene nanosheets were produced by microwave-assisted liquid-phase exfoliation. AFM measurements revealed the thicknesses of non-doped phosphorene (~4.9 nm), O-phosphorene (~5.5 nm), and N-phosphorene (~8.6 nm) [112]. The Raman spectra showed no post-treatment structural changes. The UV-vis spectra displayed characteristic peaks and blueshifts in the doped samples. XPS analysis revealed P–P and P–O bonds in all samples, with variations in the P_x_O_y_ peak intensity, indicating successful N-doping and reduced oxygen groups in N-phosphorene, further supported by the HR N 1s spectrum and XPS survey scan [111].

Table 4 offers a comprehensive summary of multiple electrocatalysts utilized in the conversion process of NO_3_^−^ into NH_3_. The catalysts listed comprise Pd-Cu/TinO_2_n^−1^, Pd-In/γ-Al_2_O_3_, 30%Cu–70%Pd, Fe_3_Ni-N-C, a single Fe atom, Co_3_O_4_ nanorod arrays, Rh/C, Cu@Th-BPYDC, RuNi-MOF, and Pd-NDs/Zr-MOF. The table provides information on the NH3 yield, FE, and electrolytes utilized. The properties and characteristics of each catalyst differentiate them, showcasing their capacity to facilitate the conversion of NO_3_^−^ into NH_3_ across diverse applications.

**Figure 4:**
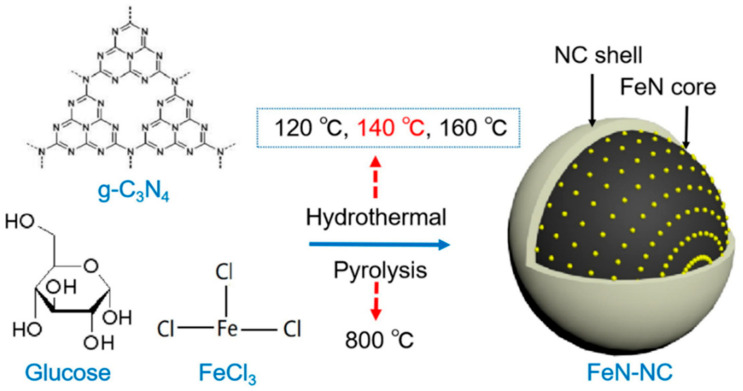
Synthetic route of nitride–carbon-encapsulated N-doped iron (FeN-NC). Adapted with permission from Wang et al. [128]. Copyright 2020, Elsevier.

The enhanced number of active sites and electronic structure of N-doping likely contribute to the superior catalytic activity of N-phosphorene. Control investigations verified that NH_3_ gas originated from the injected N_2_ gas. No NH_3_ was found in electrolytes saturated with Ar when N-phosphorene was used in copper foam (CF) [111]. Furthermore, a comparison of N-, O-, and phosphorene at various potentials revealed consistent NH_3_ yield rates (~15 μg h^−1^ mg_cat_^−1^), with N-phosphorene demonstrating superior NRR activity and HER suppression. N-phosphorene also exhibited a lower charge transfer resistance (Rct) (24.2 Ω) than phosphorene (56.3 Ω) and O-phosphorene (34.1 Ω), indicating favorable charge transfer mechanisms due to heteroatom presence [126]. Additionally, durability assessment over six consecutive electrolysis cycles at 0 V showed minimal declines in the NH_3_ yield rate and FE, confirming the stability of N-phosphorene for continuous NRR electrocatalysis. Long-term testing over 15 h confirmed the constant current density of N-phosphorene, and structural and compositional analyses after six cycles revealed negligible surface composition changes due to N_2_ adsorption during catalysis, demonstrating the remarkable stability of N-phosphorene [127]. Figure 4 shows the method for the in situ synthesis of nitrogen-doped metal materials.

### 3.3. Phosphorous-Doped Catalysts

**Figure 5:**
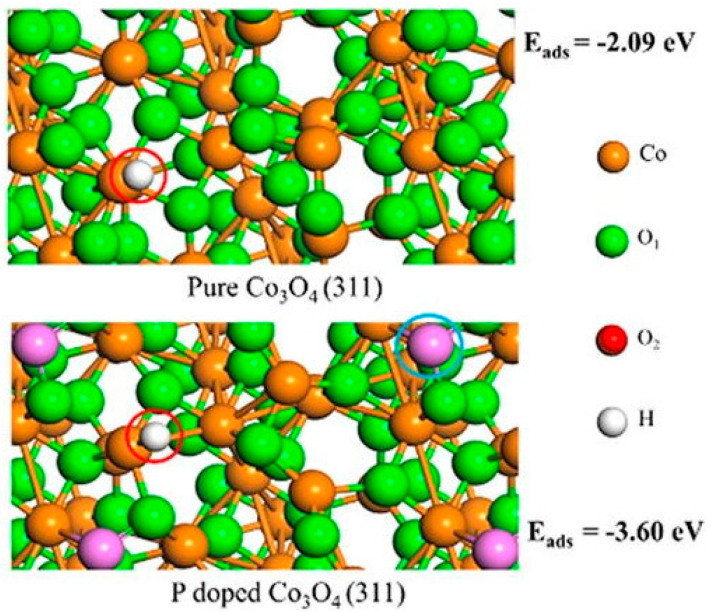
A typical phosphorus-doped catalyst. Adapted with permission from Gao et al. [130]. Copyright 2020, ScienceDirect.

Phosphorus doping often changes the crystal structure of the original catalyst, as shown in Figure 5. However, phosphorus-doped catalysts, such as phosphorus-doped potassium peroxyniobate (P-KNO), have been utilized for the electrocatalytic reduction of nitrogen to ammonia, effectively improving N_2_-to-NH_3_ conversion. P-KNO, with enriched oxygen vacancies, demonstrated an NH_3_ production rate of 23.01 μg h^−1^ mg_cat_^−1^ and an FE of 39.77% at −0.4 V vs. RHE (reversible hydrogen evolution) in a 0.1 M solution [129].

Phosphorus-doped carbon nitride, with abundant nitrogen vacancies, exhibited an outstanding NRR catalytic performance in 0.1 M Na_2_SO_4_ electrolyte, with an FE of 22.5% and an NH_3_ production of 28.67 μg h^−1^mg_cat_ ^−1^ [131]. In addition, phosphorus in the catalyst structure enhances N_2_ fixation activity and accelerates N_2_ adsorption and activation during NRRs, leading to a higher NH_3_ generation and improved selectivity [132].

The combination of phosphorus and potassium in P-KNO efficiently increased N_2_-to-NH_3_ conversion [133]. Nitrogen-doped phosphorene (N-phosphorene) with high crystallinity was synthesized using ball milling and microwave techniques. This synthesis method leads to excellent electrocatalytic performance, with an NH_3_ yield rate and FE of up to 18.79 μg h^−1^ mg_cat_^−1^ and 21.51%, respectively, at a low overpotential (0 V) compared to RHE [134].

### 3.4. Sulfur-Doped Catalysts

Sulfur-doped catalysts show promise for the electrocatalytic production of NH_3_ by reducing nitrogen. The incorporation of sulfur into diverse carbon-based materials, including graphene and porous carbon, enhances their electrocatalytic performance in ammonia synthesis via N_2_ reduction [135]. Sulfur-doped graphene (S-G) and sulfur-doped porous carbon (S-NPC) have emerged as effective and stable electrocatalysts that promote the reduction of N_2_ to NH_3_. Specifically, S-G exhibits efficacy and steadfastness in catalyzing the NRR under normal circumstances, leading to sufficient N_2_-to-NH_3_ conversion [136].

The proposed mechanism for S-doped graphene (S-G) involves the development of catalytically active sites, improved adsorption of nitrogen species, facilitated electron transfer, high NH_3_ yield, and enhanced Faradaic efficiency [135,137]. Density functional theory simulations indicate that carbon atoms adjacent to sulfur substitutions act as catalytically active sites for the NRR on S-G [135]. Incorporating sulfur increases the adsorption of nitrogen species, which is a crucial factor for the electrocatalytic NRR. By facilitating electron transmission between the reactants and electrolytes, sulfur-doped graphene reduces the energy barrier for the NRR, resulting in an accelerated reaction rate. In 0.1 M HCl, S-G exhibits exceptional performance with a substantial NH_3_ production of 27.3 μg h^−1^ mg_cat_^−1^ and a high Faradaic efficiency of 11.5% at −0.6 and −0.5 V vs. RHE, respectively, surpassing undoped graphene values (6.25 μg h^−1^ mg_cat_^−1^; 0.52%) [137]. The remarkable electrocatalytic performance of sulfur-doped graphene makes it a promising and sustainable electrocatalyst for N_2_-to-NH_3_ fixation [138].

## 4. Nitrogen Synthesis of Ammonia Using Non-Metal-Doped Catalysts

The utilization of non-metallic materials as catalysts for the N_2_ synthesis of NH_3_ represents a promising avenue for sustainable and environmentally friendly NH_3_ production methods. Therefore, as shown in Table 5, NH_3_ production via electrocatalytic nitrogen reduction presents several challenges. An example of such an issue is the rivalry between the HER and NRR. Enhancing the inhibition of HER improves the effectiveness of the NRR [139]. However, because of their sensitive catalytic structure, precious metal catalysts such as Ru and Rh show increased activity, with Ru on titanium felt enhancing NRRs and NH_3_ production [140]. Furthermore, Guo et al. found that fluorine-doped graphene demonstrates excellent performance in the oxygen reduction reaction [141], and it can be synthesized via a hydrothermal reflux method [142]. This material holds significant implications for electrocatalytic ammonia production.

## 5. Synthesizing and Activating Biomass-Derived Carbon Materials for NH_3_ Production

Biomass-derived carbon materials (BCMs) are a new type of material that possesses various catalytic characteristics, including a significant surface area and porosity. These qualities make them highly suitable for use as heterogeneous catalysts and catalytic supports. The materials exhibit compatibility with a diverse array of processes, encompassing organic transformations, electrocatalytic reactions, and photocatalytic reactions [148,149,150,151]. Biomass carbon, mostly sourced from plants, possesses the qualities of being inexpensive, abundant, eco-friendly, and sustainable, rendering it a very promising substance for electrochemical applications [152,153,154,155]. Recently created materials generated from biomass, such as porous carbons and carbon nanotubes, have superior characteristics, including a larger surface area, improved thermal stability, increased adsorption capacity, and higher electrical conductivity. These advantages are due to their small size and increased porosity.

Nevertheless, they are still very inexperienced in heterogeneous catalysis and require more refinement in order to supplant their counterparts obtained from synthetic carbon precursors [148,152,156,157,158]. Hence, the mutually beneficial association between carbon compounds produced from biomass and non-metallic-doping agents is a crucial pathway in the pursuit of environmentally friendly and high-performing materials for various uses. Biomass-derived carbons may be efficiently modified by using non-metallic-dopants such as N, P, S, and B. These dopants enhance the renewable and structurally adaptable properties of biomass-derived carbons [159]. Nitrogen-doped carbon materials, for instance, have shown exceptional promise in improving the electrochemical properties crucial for energy storage devices like supercapacitors [50,160,161].

Similarly, S-doped carbons have demonstrated heightened catalytic prowess in environmental remediation applications [135,162,163]. The comprehensive characterization of these materials, facilitated by advanced techniques such as X-ray photoelectron spectroscopy (XPS) and Raman spectroscopy, has been instrumental in elucidating the intricate structural and chemical alterations induced by doping strategies [164,165]. Li et al. made N-doped porous carbon from biomass using a basic hydrothermal–pyrolysis method. This material was very good at catalyzing reactions. The material demonstrated a significant ammonia production and FE of 16.3 μgh^−1^mg_cat_^−1^ and 27.5%, respectively [166].

Also, an electrocatalyst made from alfalfa and made up of porous carbon showed a lot of activity in the NRR, reaching an FE of 9.98% [52]. However, the carbon material that was made from bamboo shoots and used to make NH_3_ through electrochemistry only worked 27.5% of the time under normal temperature and pressure conditions in an acidic solution [166]. Therefore, Giddey et al. state that the faradaic efficiencies mentioned are insufficient for practical use. They suggest that practical needs require a minimum FE of 50% [167]. Carbon-based materials are abundant and have the unique capacity to endure acidic conditions, which makes them well suited for certain catalytic purposes [168,169,170,171,172]. This makes them very suitable for developing carbon-based catalysts without any metal content, specifically for NRRs [173,174]. Huang et al. conducted experiments that show that amorphous-oxygen-doped carbon nanosheets (O-CNs) made from biomass may be very useful as electrocatalysts for NRRs, showing great selectivity with an FE of 4.97% [175].

Similarly, in a study conducted by Wu et al., it was found that oxygen-doped hollow carbon microtubes (O-KFCNTs) derived from natural kapok fibers are a highly efficient electrocatalyst for converting N_2_ to NH_3_ without the use of metals. These microtubes exhibit excellent selectivity and achieve a high FE of 9.1% [153]. Therefore, future studies must prioritize enhancing synthesis techniques to ensure scalability and investigate the synergistic impacts of combining doping with complementary modifications to materials. Carbon materials obtained from biomass can improve performance in several multifunctional material platforms.

## 6. Conclusions and Prospects

Extensive research conducted over the years on non-metallic-doped materials for electrocatalytic NH_3_ synthesis has made considerable advancements and has the potential to solve urgent challenges in the sectors of sustainability, emission reduction, and energy transition. The incorporation of non-metallic dopants has shown encouraging outcomes in improving the electrocatalytic performance and specificity of materials. By altering various crystal structures, these dopants have demonstrated the capacity to enhance the rate of NH_3_ production, faradaic efficiency, and stability. More importantly, there has been progress in understanding the basic principles that control the electrocatalytic performance of materials doped with non-metallic elements. These basic principles have provided insights into the effects of doping on the electronic structure, surface characteristics, and reaction kinetics. This understanding serves as a strong basis for the development and enhancement in future catalysts for achieving efficient NH_3_ generation. The creation of non-metallic-doped materials for electrocatalytic NH_3_ generation is a significant advancement in sustainable and ecologically friendly synthesis processes.

Nevertheless, the electrochemical method presents the possibility of achieving carbon-neutral NH_3_ synthesis using sustainable energy sources and effective nitrate reduction. The shift towards electrocatalytic NH_3_ generation is in line with worldwide initiatives to reduce greenhouse gas emissions and move towards a sustainable energy future. In electrocatalytic NH_3_ synthesis, there are significant prospects and difficulties related to non-metallic-doped materials that need to be considered in the future. Additional investigations are required to examine other substances that can be added and their combined effects, as well as to refine the procedures used to create the substance to improve its performance and capacity to be produced on a larger scale. Thorough research is necessary to ensure the long-term stability and durability of the catalysts under actual working conditions.

Furthermore, the incorporation of catalysts doped with non-metallic elements into electrocatalytic systems that are applicable in real-world settings and the expansion of these systems to an industrial scale continue to be a crucial stage in the process of making them commercially viable. Effective collaboration among academics, industry, and policymakers is essential in addressing the technological, economic, and regulatory factors necessary for the effective deployment of electrocatalytic NH_3_ synthesis. The advancements achieved in non-metallic-doped materials for electrocatalytic NH_3_ generation represent a promising approach for achieving sustainable NH_3_ synthesis. These catalysts have the potential to transform the NH_3_ manufacturing business and contribute to a more environmentally friendly and sustainable future by addressing important issues, such as sustainability, emission reduction, and energy transition.

## Figures and Tables

**Table 1:** Summary of post-synthesis doping methods.

Post-Synthesis Doping Method	Source of Non-Metal	Material Doped	Temperature Range (°C)	Time	Result	Ref.
In situ electron-beam irradiation	Paraffin wax	Boron nitride nanotubes	-	-	A well-controlled, little-damaging, simple strategy	[64]
Direct nitrogen reaction	Nitrogen and cyanamide	Single-walled carbon nanotubes/graphene sample	500–800 °C	1 h	Improved catalytic properties	[65]
Thermal-diffusion-based doping	Boron from boric acid	Silicon quantum dots	400–600 °C	1 h	Network-interconnected Si particles.	[66]
Ethylene amine vapor doping	Ethylene amine	Graphene	300 °C	1 h	High-performance transparent electrodes	[67]
Solvothermal method	Nitrogen from melamine	Carbon nanoparticles from CCl_4_	-	-	Low-cost alternative to commercial Pt/C catalysts	[68]
Solid template approach	Nitrogen from melamine	core–shell SiO_2_@RF composites	600 and 900 °C	1 h	High specific surface areas and thermal stabilities	[69]

**Table 2:** Synthesis methods for in situ-N-doped carbon compounds.

Synthesis Technique	Description	Source/Product	Ref.
CVD	Simultaneous synthesis of carbon material and nitrogen doping.	N-doped carbons	[79]
Infiltration and subsequent CVD	A novel approach involving Y zeolite infiltration with an ionic liquid and subsequent CVD. Co-doped microporous carbon exhibited pyridinic, pyrrolic, and graphitic nitrogen.	Co-doped microporous carbons	[70]
CVD	Utilization of C_2_H_4_/NH_3_ combinations in CVD for creating N-doped carbon nanofibers (N-CNFs)	N-CNFs	[80]
Carbonization	N-doped carbon material preparation from polyaniline (PANI) through carbonization at 800 °C using various salt precursors	Synthesis from PANI	[73]
Carbonization	Employing imidazolium/pyridinium ionic liquids (ILs) for N-doped carbon synthesis	N-doped carbons	[81]
Electrospinning, carbonization	Creation of hollow-particle-based N-doped carbon nanofibers (N-CNFs) using PAN and ZIF-8, followed by carbonization.	Hollow-particle-based N-CNFs	[82]
Hydrothermal carbonization (HTC)	Hydrothermal carbonization (HTC) of biomass for creating N-doped carbon compounds.	Biomass-derived N-doped carbon	[83]
Heat treatment, chemical activation	Closed porosity and low surface area in resulting hydrochars necessitating additional treatment.	Biomass-derived N-doped carbon	[71]
Pyrolysis	Conventional pyrolysis of biomass for producing N-doped carbon nanosheets without chemical activation.	Advanced pyrolysis for N-doped carbon Nanosheets	[84]

**Table 3:** Summary of synthesis methods for metal catalysts supported on N-doped carbon materials.

Methodology	Description	Reference
Capping-agent-protected NPs	Uses capping agents for NP growth regulation; concerns about the negative impact on catalytic activity.	[101,102]
N-doped carbon supports	Achieves small, evenly distributed NPs. Nitrogen functionalities enhance catalytic performance.	[103,104]
Synthetic techniques for NPs on N-doped carbon	Three categories: loading on pre-synthesized N-doped carbon, simultaneous incorporation, and in situ synthesis.	[83,105]
Conventional techniques for loading NPs	Includes impregnation, deposition–precipitation, and sol immobilization with subsequent reduction.	[106,107]
In situ synthesis of NPs on N-doped carbon	The pyrolysis step allows the simultaneous incorporation of metal and nitrogen.	[107,108]
Use of MOF as a sacrificial template	MOFs are used as templates for intriguing metal/N–carbon compositions.	[109,110]

**Table 4:** Summary of electrocatalysts for the reduction of NO_3_^−^ to NH_3_.

Catalysts	NH_3_ Yield	FE (%)	Electrolyte	Year Published	Reference
Pd-Cu/TinO_2_n^−1^	-	22	1 mM NaNO_3_	2018	[113]
Pd–In/γ-Al_2_O_3_	-	71.5	1.4 mM NO_3_^−^	2009	[114]
Pt	-	49	3000 mg·L^−1^ NO_3_^−^	2006	[115]
30%Cu-70%Pd	-	58	0.05 M KNO_3_	2009	[116]
Fe_3_Ni-N-C	Nitrate-to-N_2_	99.3	100 ppm NO_3_^−^	2021	[117]
Single Fe atom	0.46 mmol·h^−1^·cm^−2^	75	0.50 M KNO_3_	2021	[118]
Co_3_O_4_ nanorod arrays	0.854 mmol·h^−1^·cm^2^	33.6	100 g L^−1^ NO_3_^−^	2019	[119]
Rh/C	34.4 μg h^−1^cm^−2^	20.8	0.1 M KNO_3_	2021	[120]
Cu@Th-BPYDC	225.3 μmolh^−1^cm^−2^	92.5	100 mM KNO_3_	2022	[121]
RuNi-MOF	274 mgh^−1^mg_cat_^−1^	73	50 mg L^−1^ NO_3_^−^	2022	[122]
Pd-NDs/Zr-MOF	287.31 mmol·h^−1^·g_cat_^−1^.	58.1	500 ppm NO_3_^−^	2022	[123]
Pd cuboctahedrons	306.8 μgh^−1^mg_cat_^−1^	35	20 mM NO_3_^−^	2021	[124]
PTCDA/O-Cu	436 μg h^−1^cm^−2^	85.9	500 ppm NO_3_^−^	2020	[125]

**Table 5:** Types and characteristics of electrocatalyst for N_2_ synthesis of NH_3_.

Catalyst Type/Process	Characteristics	Challenges and Limitations	Ref.
Metallic catalysts (Ru, Rh)	Ru electroplated on titanium felt enhances NH_3_ production; Ru on porous graphite improves NRR	Catalytic structure sensitivity; cost and scarcity of precious metals	[139,140]
Monatomic catalysts	Strong chemical interaction with nitrogen; outstanding activity	High cost and scarcity for large-scale application	[143,144]
Non-precious metal catalysts	Low Faraday efficiency; activation via sp hybrid orbitals	Limited Faraday efficiency despite low cost	[145]
Metal-free catalysts (N, P)	Carbon-based compounds doped with N and P; PCN-NVs increase ammonia yield and Faraday efficiency	Cost and scarcity challenges for large-scale use	[78]
Perovskite catalysts	LaFeO_3_ effective in oxidizing N_2_; NTP enhances current density; Cu/CuO catalyst reduces NOx−	Gibbs free energy spectrum investigation; challenges in N_2_ activation	[146,147]

## Nomenclature

NV	Nitrogen vacancies
B	Boron
P	Phosphorus
S	Sulfur
NH_3_	Ammonia
NO_3_^−^	Nitrate
MOFs	Metal–organic frameworks
HER	Hydrogen evolution reaction
CVD	Chemical vapor deposition
HTC	Hydrothermal carbonization
ORR	Oxygen reduction reaction
GO	Graphene oxide
NG	Graphene sheets
N-RGO	N-doped reduced graphene oxide
CNTs	Carbon nanotubes
N-CNFs	N-doped carbon nanofibers
NRRs	Nitrogen reduction reactions
HER	Hydrogen evolution reaction
FE	Faradaic efficiency
O-KFCNTs	Hollow carbon microtubes
NMP	N-methyl-2-pyrrolidone
g	C_3_N_4_ porous polymeric carbon nitride
C_2_H_4_N_4_	Dicyandiamide
C_3_H_7_NO	Dimethylformamide
C_3_H_6_N_6_	Melamine
BCMs	Biomass-derived carbon materials
